# Identification of Key Uric Acid Synthesis Pathway in a Unique Mutant Silkworm *Bombyx mori* Model of Parkinson’s Disease

**DOI:** 10.1371/journal.pone.0069130

**Published:** 2013-07-24

**Authors:** Hiroko Tabunoki, Hiromasa Ono, Hiroaki Ode, Kazuhiro Ishikawa, Natsuki Kawana, Yutaka Banno, Toru Shimada, Yuki Nakamura, Kimiko Yamamoto, Jun-ichi Satoh, Hidemasa Bono

**Affiliations:** 1 Department of Bioinformatics and Molecular Neuropathology, Meiji Pharmaceutical University, Tokyo, Japan; 2 Database Center for Life Science (DBCLS), Research Organization of Information and Systems (ROIS), Tokyo, Japan; 3 The Center of Genetic Resources, Kyushu University, Fukuoka, Japan; 4 Department of Agricultural and Environmental Biology, Graduate School of Agricultural and Life Sciences, The University of Tokyo, Tokyo, Japan; 5 Insect Genome Research Unit, National Institute of Agrobiological Sciences, Tsukuba, Japan; Hertie Institute for Clinical Brain Research and German Center for Neurodegenerative Diseases, Germany

## Abstract

Plasma uric acid (UA) levels decrease following clinical progression and stage development of Parkinson’s disease (PD). However, the molecular mechanisms underlying decreases in plasma UA levels remain unclear, and the potential to apply mutagenesis to a PD model has not previously been discovered. We identified a unique mutant of the silkworm *Bombyx mori* (*B.mori*) *op*. Initially, we investigated the causality of the phenotypic “*op”* by microarray analysis using our constructed KAIKO functional annotation pipeline. Consequently, we found a novel UA synthesis-modulating pathway, from DJ-1 to xanthine oxidase, and established methods for large-scale analysis of gene expression in *B. mori*. We found that the mRNA levels of genes in this pathway were significantly lower in *B. mori op* mutants, indicating that downstream events in the signal transduction cascade might be prevented. Additionally, levels of *B.mori* tyrosine hydroxylase (TH) and DJ-1 mRNA were significantly lower in the brain of *B. mori op* mutants. UA content was significantly lower in the *B. mori op* mutant tissues and hemolymph. The possibility that the *B. mori op* mutant might be due to loss of DJ-1 function was supported by the observed vulnerability to oxidative stress. These results suggest that UA synthesis, transport, elimination and accumulation are decreased by environmental oxidative stress in the *B. mori op* mutant. In the case of *B. mori op* mutants, the relatively low availability of UA appears to be due both to the oxidation of DJ-1 and to its expenditure to mitigate the effects of environmental oxidative stress. Our findings are expected to provide information needed to elucidate the molecular mechanism of decreased plasma UA levels in the clinical stage progression of PD.

## Introduction

Parkinson’s disease (PD) is a common neurodegenerative disorder with an etiology involving oxygen radicals and other oxidants that attack dopaminergic neuronal cells and which damage and deplete dopamine levels [1]. Genetic studies have identified 18 genes associated with PD at different loci based on family linkage analysis (PD; Online Mendelian Inheritance in Man (OMIM) 168600). PD-associated gene knockout animal models have been developed as familial PD models [2].

The majority of idiopathic PD cases, however, are the result of sporadic onset caused by environmental stress [3], and a molecular-based mechanism of oxidative stress has been developed. In animal models of sporadic PD, oxidative stress has been simulated using parkinsonian neurotoxins that are mitochondrial complex I inhibitors [4], namely 1-methyl-4-phenyl-1,2,3,6-tetrahydropyridine (MPTP), 6-hydroxy-dopamine (6-OHDA), paraquat (PQ) and rotenone (ROT).

The final product of purine metabolism, uric acid (UA), plays an important role as a physiological antioxidant [5]. In recent years, several groups have reported the correlation between decreased plasma UA levels and neuron cell failure in the substantia nigra, clinical progression and stage of PD [6], [7], [8], [9], [10]. Conversely, high plasma UA concentrations in hyperuricemia may reduce the risk and delay the progression of PD [11]. Plasma UA might be expended to resist oxidative injury in PD, but the molecular mechanism underlying the decrease in plasma UA in advanced clinical stages of PD has not been analyzed using either of these models.

Here, we used a silkworm *B. mori* mutant with reduced levels of UA to examine the mechanisms involved in UA metabolism. In silkworms, UA is mainly synthesized in the fat body, from where it is transported to the integument via the hemolymph. On the other hand, UA is eliminated through the Malpighian tubules. UA accumulates as urate granules, which cause a whitening of the integument color [12]. UA is typically the ultimate metabolite in insects, but in *B. mori* UA is partly converted to urea by urate oxidase [13]. Mutant larvae unable to synthesize UA due to a deficiency in xanthine oxidase (XD/XO) [14], [15], [16] or failure of the UA transporter [17], cannot accumulate UA in the larval epidermis and take on a translucent appearance.

**Figure 1:**
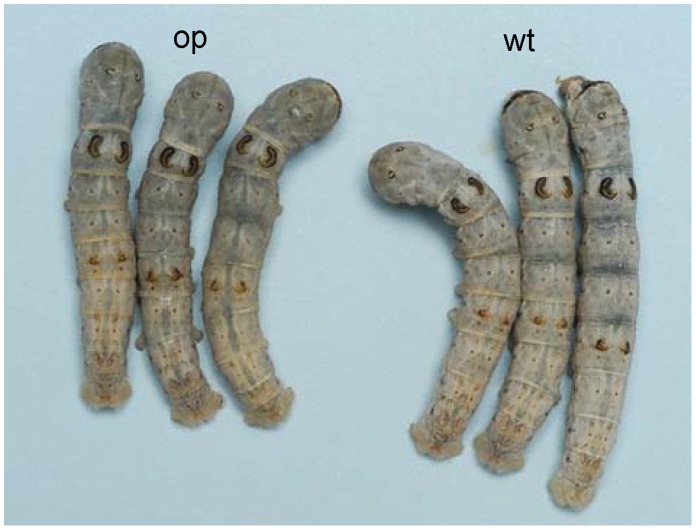
Phenotypes of *op* and wild-type larvae. Left, *op* mutants (*op*), which have a translucent integument. Right, wild-type larvae (wt), which have a white integument.

The *B. mori op* mutant exhibits spontaneous and pronounced translucency during the larval stage (Fig. 1) and occasional unique actions such as vibration (Video S1).

Classical linkage analysis has demonstrated that a mutation located on chromosome 23 is responsible for the extraordinarily high phenotype mortality, particularly in the pupal stage, as well as male infertility (NBRP silkworm database: http://www.shigen.nig.ac.jp/silkwormbase/). Despite the unique phenotypes of the *op* mutant with reduced levels of UA, the *op* causative gene and position has not been clarified.

In the present study, we characterized the *B. mori op* mutant and identified the novel uric acid synthesis pathway using microarray analysis.

## Results

### Screening for Target Molecules using Microarray

We investigated the number of human homologs in the *B. mori* genome. We identified 8096 human homologs among 14,623 total transcripts in the *B. mori* consensus gene set by merging all the gene sets using GLEAN (http://sgp.dna.affrc.go.jp/pubdata/genomicsequences.html). Furthermore, enrichment analysis of the human homologs (Table S1) showed that the conserved human homologs in *B. mori* that showed the most statistically significant metabolic pathways were those for spliceosome (p = 1.05E-13), pyrimidine metabolism (p = 2.16E-08) and purine metabolism (p = 3.01E-08).

**Figure 2:**
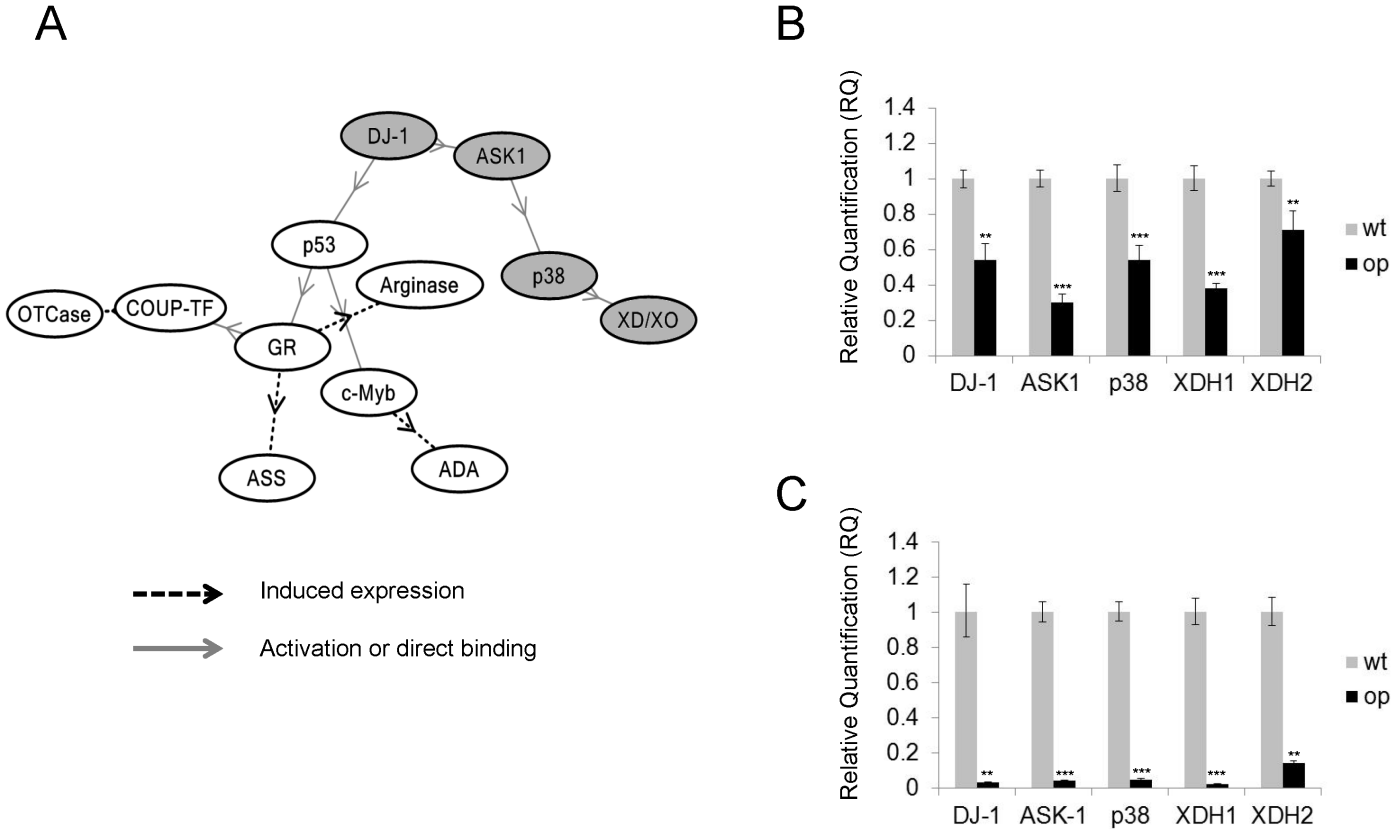
Microarray screening and quantitative RT-PCR confirmation of genes that are down-regulated in *op*. (**A**) Molecular network generated by KeyMolnet for genes showing variation in *op*. Molecular relations are indicated by solid lines with arrows (direct binding or activation) or broken lines with arrows (induced expression). The pathway from DJ-1 to XD/XO is highlighted in gray. Relative mRNA expression in (**B**) fat body and (**C**) testis in wild-type and *op* mutant as Relative Quantification (RQ) values. RQ represents the relative expression level compared to the reference sample. Error bars represent the relative minimum/maximum expression levels about the mean RQ expression level. (B) DJ-1(p = 0.0038), ASK1(p = 0.0005), p38(p = 0.0004), XDH1(p = 0.0009), XDH2(p = 0.0011), (C) DJ-1(p = 0.0008), ASK1(p<0.001), p38(p<0.001), XDH1(p = 0.0009), XDH2(p = 0.0075).

Microarray analysis was performed to investigate the causality of the phenotype of *op*. We used TIBCO Spotfire software (TIBCO, Palo Alto, CA, USA) for the identification of differentially expressed probe sets between op and wild-type larvae, for the calculation of correlation coefficients, and for bi-directional hierarchical clustering using Ward’s method (Figure S1). Next, we imported the list of Kaiko array IDs for these genes and their expression levels into KeyMolnet. KeyMolnet generated a molecular network, presenting a significant relationship for the pathway of purine metabolism (score = 177.109 with P-value = 4.841E-054) and transcriptional regulation by p53 (score = 78.113 P-value = 3.059E-024). In further exploration of associations between the genes involved in purine metabolism and transcriptional regulation by p53 using KeyMolnet, we detected the unique pathway (score = 14.888 with the P-value = 3.299E-005) from DJ-1 to xanthine dehydrogenase/xanthine oxidase (XD/XO) (Fig. 2A; highlighted in gray).

Several target molecules related to this pathway (Fig. 2A; highlighted in gray) were validated by qRT-PCR. *B. mori* has two genes encoding xanthine dehydrogenase, XDH1 (Gene ID: 692751) and XDH2 (Gene ID: 692757), whichare orthologous to human XD/XO [15]. In this pathway, DJ-1, apoptosis signal-regulating kinase 1 (ASK-1), p38 mitogen-activated protein kinase (MAPK) and XDH1 and XDH2 mRNA were significantly decreased in the fat body (Fig. 2B) and testis (Fig. 2C) of the *op* larval mutant.

### Levels of TH and DJ-1 mRNA and TH Immunohistochemistry in op Larval Brain

**Figure 3:**
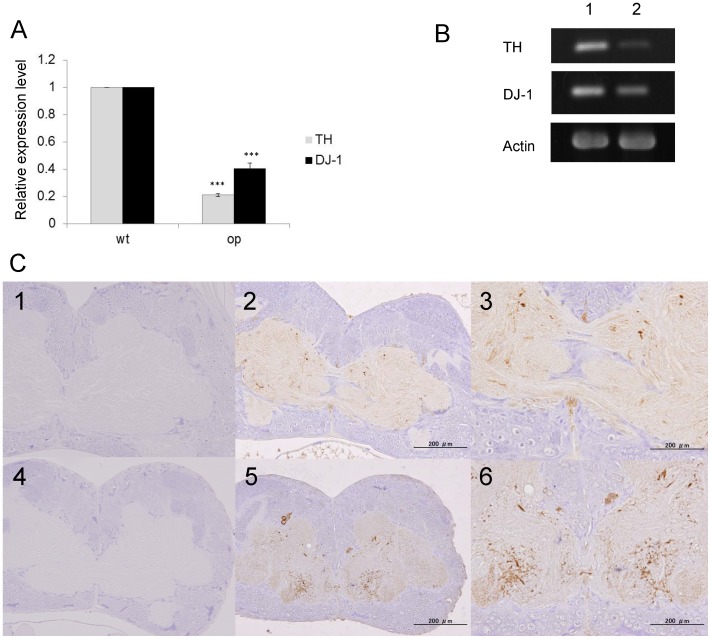
*B.* *mori* TH and DJ-1 mRNA expression in brain of wild-type and *op* mutant larvae by RT-PCR. (**A**) mRNA expression levels were measured by Image J ver. 1.37 c, and plotted to compare *op* and wild-type mRNA levels (TH; P<0.0001, DJ-1; P = 0.0017). (**B**) Lane 1, wild-type; lane 2, *op* mutant. (**C**) **Immunohistochemical localization of tyrosine hydroxylase (TH) in** ***op*** **and wild-type brain.** Tissue sections were prepared from day 5 to 7 fifth instar larvae. 1 (*op*) and 4 (wild-type) are negative controls. TH positive regions (2 and 3:*op*, 5 and 6:wild-type) are stained brown. Bar: 200 µm.

Tyrosine hydroxylase plays (TH) an important role in the biosynthesis of dopamine andproduction is reduced by a decrease in TH expression, leading to the onset of PD [18]. Thus, TH expression levels are used for diagnosis of PD. To investigate the expression levels of *B. mori* TH and DJ-1 mRNA in fifth instar *op* mutant and wild-type larval brain, we performed reverse transcription polymerase chain reaction (RT-PCR) and immunohistochemistry. *B. mori* TH and DJ-1 mRNA levels were found to be significantly lower in the brains of *op* larval mutants (Fig. 3A and B, Figure S2).

Also, the TH-positive region was decreased in the brain of *B. mori op* larvae (Fig. 3C, 1–3) compared to wild-type larvae (Fig.3C, 4–6) by immunohistochemistry.

**Figure 4:**
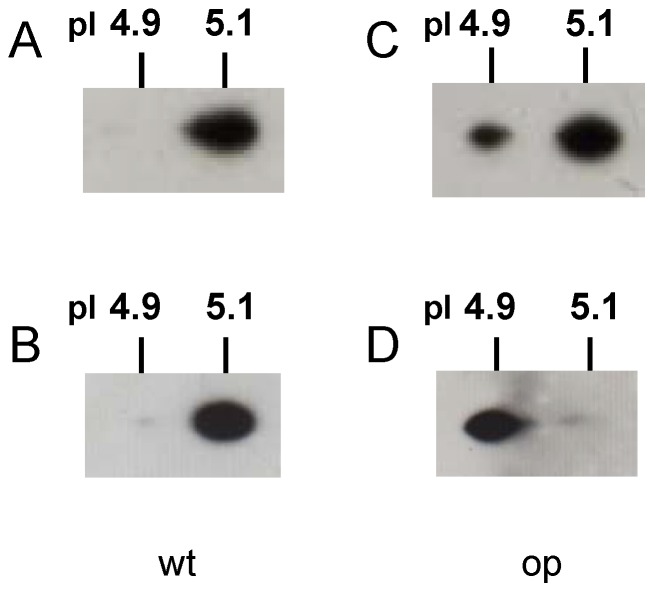
Analysis of oxidized DJ-1 protein in *op* and wild-type larvae. (**A**) and (**B**) show the wild-type, (**C**) and (**D**) show the *op* mutant. Oxidized DJ-1 was determined by 2D-PAGE and immunoblotting. Upper panel, fat body; lower panel, testis.

**Figure 5:**
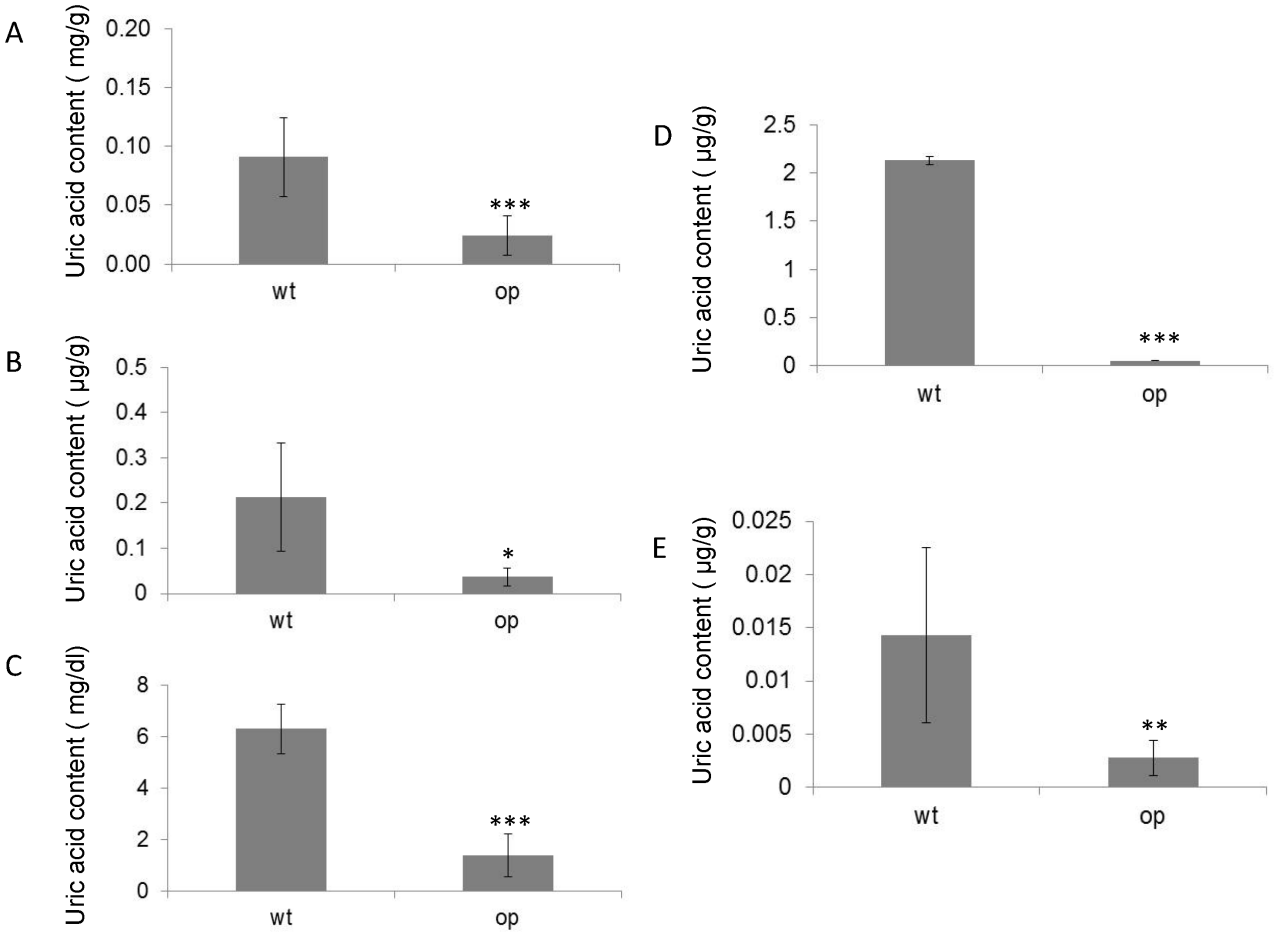
Uric acid content in wild-type and *op* larve. Samples were collected from wild-type and *op* larvae and Uric acid was extracted and measured in (**A**) integument (P<0.001), (**B**) fat body (P = 0.037), (**C**) hemolymph (P = 0.0002), (**D**) Malpighian tubule (P<0.0001) and (**E**) testis (P = 0.004).

**Figure 6:**
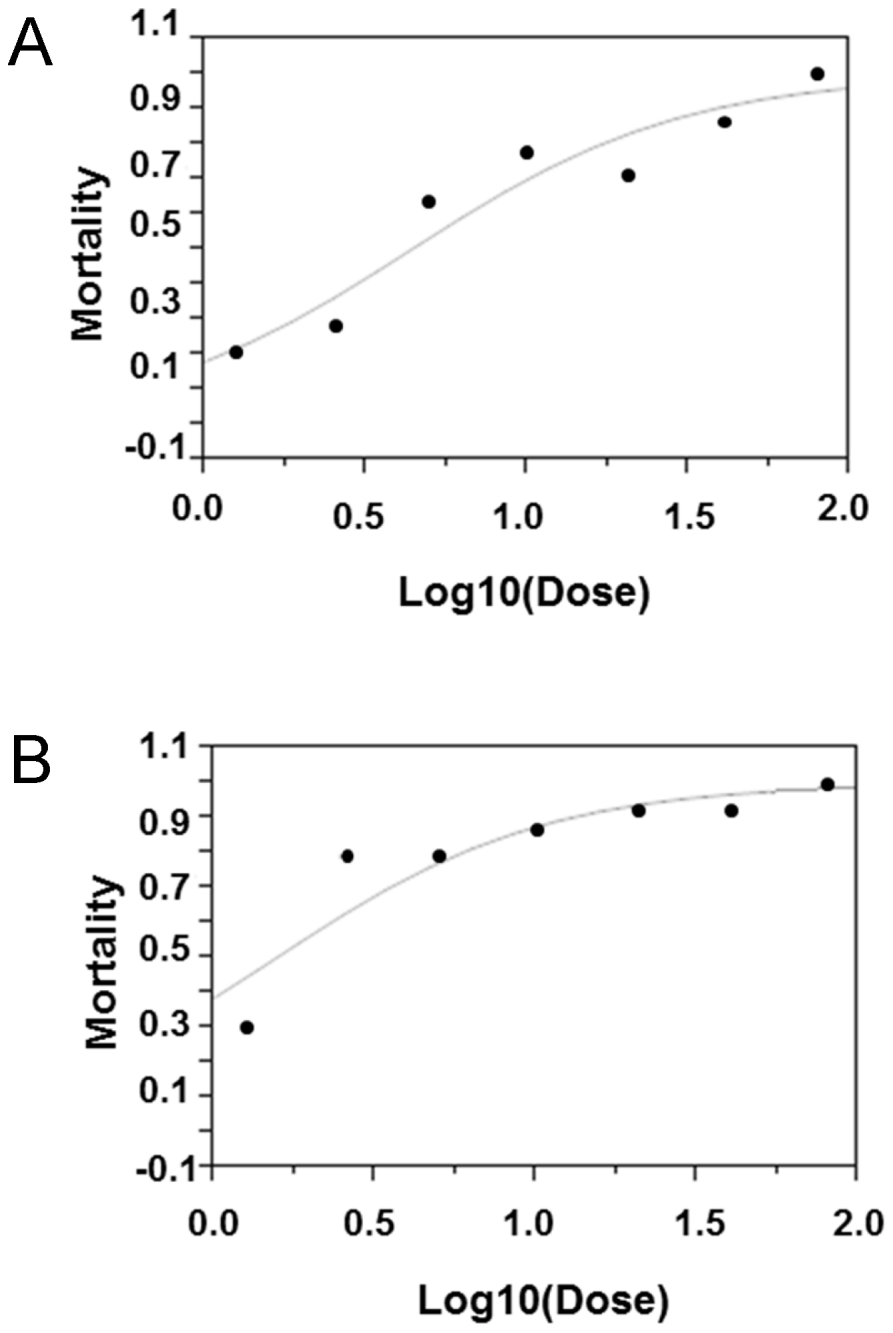
Dose mortality curve of *op* and wild-type larvae exposed to rotenone. (**A**) wild-type, (**B**) *op* mutant. The vertical axis shows the mortality and the horizontal axis shows the rotenone Log_10_ dose. Solid circles represent observational data.

### Detection of the Oxidized form of DJ-1

To determine whether the oxidized DJ-1 is present in *B. mori*, testis and fat body dissected from *op* or wild-type silkworms were examined. The fatbody (Fig. 4A) and testis (Fig. 4B) DJ-1 from wild-type larvae had a pI in the non-acidic range (pI, 5.1; non-oxidized form) by 2D-PAGE and immunoblotting. In contrast, the fat body (Fig. 4C) and testis (Fig. 4D) from *op* mutants produced a shift in pI to the acidic range (pI, 4.9; oxidized form) by 2D-PAGE and immunoblotting. Therefore, the oxidization of *B. mori* DJ-1 occurs only in the *op* mutant.

### UA Content was Decreased in op Mutants

UA content was measured in the integument, hemolymph, fat body and Malpighian tubule of wild-type and *op* larvae. UA content was significantly decreased in all samples of *op* compared to wild-type larvae (Fig. 5A–E).

### Op Mutant has Decreased Resistance to Rotenone-induced Oxidative Stress

ROT was used to examine the resistance to oxidizing stress in *op* larvae. The 50% lethal dose (LD_50_) of ROT for day 3 fifth instar larvae was determined to be 4.3 µg/g (95% CI, 1.5–52.5; Fig. 6A) for wild-type and 1.6 µg/g (95% CI, 0.8–13.0; Fig. 6B) for *op* larvae. The LD_50_ was significantly lower for *op* larvae than for wild-type larvae.

## Discussion

In this study, we characterized the *B. mori op* mutant, one of the *B. mori* translucent larval skin mutants.

To investigate the causative factorsof phenotype *op* mutant, we employed microarray analysis using our constructed KAIKO functional annotation pipeline. Microarray analysis has shown efficacy for detecting many causative factors related to phenotypic *op* despite the fact that the *op* causative gene has yet to be clarified.

Here, we found a non-canonical pathway that is involved in the UA synthesis pathway from DJ-1 to XD/XO (Fig. 2A; highlighted in gray).

ASK-1 is a member of the mitogen-activated protein kinase family that activates the MKK3/MKK6-p38 signaling pathway [19]. P38 kinase belongs to the MAP kinase family and can be activated by a wide variety of stressors, while XD/XO is phosphorylated through a signaling pathway that involves p38 kinase [20]. XD/XO oxidizes hypoxanthine to xanthine, and then xanthine to UA. Most of the genes identified by KeyMolnet analysis were oxidative stress-responsive genes. In addition, DJ-1, ASK-1, p38 and two XD/XOs mRNAs were significantly down-regulated in *op* mutants.

We focused on DJ-1 for two reasons: DJ-1 is the initiator of this pathway, and the function of DJ-1 varies with the degree of oxidative stress [21].

Next, we examined TH and DJ-1 mRNA levels in the brain of *B. mori op* larvae. Interestingly, *B. mori op* larvae showed significantly lower mRNA levels of TH and DJ-1. While, the number of TH-positive neurons did not change in the DJ-1 deficient mouse model [22], [23], DJ-1 silencing by small interference RNA (siRNA) results in decreased TH expression in the human dopaminergic CHP-212 cell line [24].

In the case of expression level of mRNA between *B. mori*, DJ-1 and TH might share certain traits with human.

Moreover, oxidized *B. mori* DJ-1 was only present in the fat body and testis of the *op* larvae, while almost all of the *B. mori* DJ-1 in the testis was present in the oxidized form.

It is known that the isoelectric point (pI) of DJ-1 shifts to acidic (the oxidized form) during exposure to oxidative stress. Findings from our previous study also showed that *B. mori* DJ-1 in BmN4 cells changes to an oxidized form that is affected by ROT treatment, indicating its susceptibility to oxidative stress [25].

Human DJ-1 is induced by oxidative modification and is rapidly oxidized at position Cys 106 [26]; DJ-1 appears to directly scavenge free radicals from mitochondria in response to many different types of oxidative stress. DJ-1 produced by the oxidation of Cys 106 plays an important role in resistance to oxidative stress. However, under high levels of oxidative stress, DJ-1 is transformed to a more acidic form and becomes less resistant to oxidation; its subsequent degradation promotes the development of PD [21].

*B.mori* DJ-1 has two cysteines at residues 46 and 106 from the N-terminal methionine [25]. However, we could not verify the oxidative modification of Cys 106 in the acidic form of *B.mori* DJ-1. Our current study might suggest that DJ-1 in the *B. mori op* larvae had not only lost its function but also depressed mRNA expression due to prolonged exposure to environmental stress. Furthermore, expression of ASK1 in *B. mori* is decreased by exposure to strong UV irradiation [27]. We also confirmed that mRNA levels of DJ-1, ASK1, p38 and two XD/XOs were decreased by exposure to strong UV irradiation in day 3 of fifth instar larvae of wild-type *B. mori* (data not shown). Hence, these environmental stressors may disrupt the signal transduction cascade.

UA content was significantly decreased in the integument, fat body, hemolymph, Malpighian tubule and testis in *B. mori op* mutants, suggesting that UA synthesis, transport, elimination and accumulation are all decreased by the loss of BmDJ-1 function and environmental oxidative stress in *B. mori op* mutants.

UA directly scavenges oxygen radicals and produces allantonin and allantonic acid, which confer resistance to oxidative damage in humans [28]. Furthermore, UA plays a protective role against photooxidative stress in *B. mori*, as evidenced by the significantly reduced survival rate in larvae under UV irradiation with exposure to allopurinol, an inhibitor of UA synthesis [29]. It has been reported that a 6-OHDA-induced decrease of GSH (Glutathione-SH) and SOD (Superoxide dismutase) was partially reversed by addition of urate [30]. This result may suggest that urate, GSH and SOD were cooperated in protection against 6-OHDA-induced cell injury in PC12 cells.

Our results show that the *op* larvae are 63% less resistant to ROT-induced oxidative stress than the wild-type larvae.

Thus, in the case of the *B. mori op* mutants, the relatively low availability of UA is due to the oxidation of *B. mori* DJ-1 with this small amount being expended to mitigate the effects of environmental oxidative stress. Consequently, the phenotype of the *B. mori* mutant *op* larvae is characterized by translucent skin due to the oxidation of *B. mori* DJ-1 caused by a vicious cycle that has the potential to exacerbate the damage resulting from oxidative stress.

Low DJ-1 levels and the oxidized form of DJ-1 in spermatozoa are known to cause male infertility in humans [31]. In our data, the expression levels of *B. mori* DJ-1 protein were decreased (data not shown) and DJ-1 changed to the oxidized form in the testis of fifth instar *op* mutant larvae. Taken together, these observations are consistent with the phenotypic characteristics of *B. mori op* mutants observed in the present study.

Due to the lack of adequate animal models, the function of these molecules in PD pathogenesis is poorly understood. In addition, lower plasma UA levels in DJ-1 mutant PD patients or in other animal models have not been reported to date. We speculate that the *B. mori* DJ-1 gene is located in scaffold 2995719-2998746 of chromosome 23, suggesting that the *B. mori* DJ-1 protein might control oxidative stress in the cell due to nitric oxide (NO) and serve as a development modulation factor in metamorphosis [25]. Human DJ-1 is a causative gene of PARK7-linked familial PD [32] and plays a role in resisting oxidative stress as well as being related to male infertility in human studies [33], [34], [35]. Hence, the gene responsible for *op* might be *B. mori* DJ-1, which would be consistent with several phenotypic characteristics of *B. mori op* mutants.

In future studies, we aim to investigate whether BmDJ-1 alone regulates the phenotype of the *B. mori op* mutant and also to further elucidate the advanced molecular mechanisms in our identified pathway using an RNAi approach *in vitro* and *in vivo* and investigate relationships with other anti-oxidant enzymes such as GSH and SOD.

In summary, characterization of the *B. mori op* mutant with its unique phenotype shows that it has reduced levels of UA. Using microarray analysis of the *B. mori op* mutant, we identified a key pathway in UA synthesis. This is the first report indicating the possible involvement of DJ-1 in the initiation of UA synthesis at the molecular level. UA appears to be present in lower quantities due to the presence of dysfunctional DJ-1, in addition to being expended to limit oxidative stress. Our findings may facilitate a better understanding of the molecular mechanisms behind decreased plasma UA levels and clinical stage progression of PD.

## Methods

### Ethics Statement

The study protocol for the experimental use of animals to minimize animal suffering was approved by the Ethics Committee of Meiji Pharmaceutical University, Japan (Approval ID 2302).

### Insects

*B. mori* strain o751 (*op* and wild-type), maintained at the Institute of Genetic Resources, Faculty of Agriculture, Kyushu University (NBRP silkworm database: http://www.shigen.nig.ac.jp/silkwormbase/) was used in all experiments. Silkworms were reared on fresh mulberry leaves and kept at 25°C on a 12-hour light/12-hour dark cycle.

### Microarray Analysis

Total RNA was isolated from testis of day 3 fifth instar wild-type and *op* larvae using RNeasy Mini Kits (Qiagen, Valencia, CA, USA) and quantified on an Agilent 2100 Bioanalyzer (Agilent Technologies, Palo Alto, CA, USA). Microarrays of Cy3-labeled cRNA were prepared for *op* and wild type (n = 5 each). Briefly, 300 ng total RNA was processed using an Agilent Quick Amp Labeling Kit (Agilent Technologies) according to the manufacturer’s instructions followed by purification by an RNeasy Mini Kit (Qiagen) with quantification on an Agilent 2100 Bioanalyzer (Agilent Technologies) and a NanoDrop 1000 spectrophotometer (Thermo Scientific, Waltham, MA, USA). Hybridization was performed using a Gene Expression Hybridization Kit (Agilent Technologies) for which 1.65 µg of Cy3-labelled cRNA was mixed, fragmented, and hybridized to a silkworm 4×44 K custom oligo-microarray slide containing 43,864 spots of 60-mer oligonucleotides constructed from 17,615 EST silkworm sequences (Agilent Technologies) and incubated at 65°C for 17 h with shaking at 10 rpm. For screening, two wild-type and two *op* samples were applied to a single array slide. The array was washed in Agilent Gene Expression Wash Buffer 1 (Agilent Technologies) for 1 min at room temperature and Agilent Gene Expression Wash Buffer 2 (Agilent Technologies) for 1 min at 37°C. Intensities of the hybridized probes were detected with an Agilent G2565BA Microarray Scanner (Agilent Technologies) with 5-µm scan resolution, and the signals were extracted with G2565AA Feature Extraction Software v. 9.5 (Agilent Technologies). The raw data collected in text files were normalized ‘per spot’ and ‘per chip’ using the GENESPRING GX v. 10.3 program (Agilent Technologies). The microarray data discussed in this publication have been deposited in NCBI’s Gene Expression Omnibus (GEO) database and are accessible through GEO accession numbers GSM926580, GSM926581, GSM926582 and GSM926583 (series GSE37735).

### KAIKO Functional Annotation Pipeline for Molecular Network Analysis

In order to functionally annotate silkworm genes, silkworm genes homologous to human genes were identified by conducting a systematic BLAST search (tblastx) with a cut-off E-value of significant homology at 1e-10 (query: silkworm cDNA sequence; database: whole human cDNA sequence set from Ensembl database). However, because *B. mori* does not have a formal gene ID due to the lack of functional annotation for *B. mori* genes, we used the Kaiko array ID number for the assignment table. KeyMolnet could not recognize the Ensembl transcription ID, so we converted the human Ensembl transcription ID to Uniprot ID. The Kaiko array ID both with Ensemble transcription ID and Uniprot ID annotations (12,981 in Kaiko array ID, 28.7% of total) were used for further analyses (Table S3). Using the generated assignment table, conserved pathways common to silkworms and humans were reconstructed by projecting silkworm genes onto the human pathway.

### Molecular Network Analysis

Molecular network analysis was performed using KeyMolnet software (version 5.6 I, IMMD Inc., Tokyo, Japan) in which known molecular data have been curated by expert biologists [36]. By importing the list of molecule IDs and gene expression values from microarray data, KeyMolnet automatically generates the corresponding molecules as nodes on a network. We converted the molecular IDs from the silkworm array to human UniProtKB IDs, utilizing the assignment table described above and then input the list of genes obtained from the microarray data into this software to yield the significant molecular networks with corresponding E-values.

### Quantitative RT-PCR

To quantify RNA expression levels, total RNA was extracted from pooled testis and fat body tissue dissected from day 3 fifth instar larvae (n = 5 each) using an RNeasy Mini Kit (Qiagen) separately from the extractions for the array experiments. One-step RT-PCR was performed in 20-µl reaction volumes using 1 µg of RNA template and custom-made primers and probes (Table 1) with a TaqMan RNA-to-CT 1-Step Kit according to the manufacturer’s instructions (Applied Biosystems, Foster City, CA, USA). Quantitative RT-PCR (qRT-PCR) was performed on a 7500 Fast Real-Time PCR system (Applied Biosystems) following the Delta-Delta Ct method. Actin was utilized as an endogenous reference against which RNA expression levels were standardized, and all data were calibrated against universal reference data. All assays were performed in triplicate.

**Table 1:** Primers and probes used for quantitative RT-PCR.

Gene	Probe	Forward primer	Reverse primer
ASK1	ACGACAAATCCGGCGGCCAC	5′-TTGACGGACGTCGGAACA-3′	5′-GCAGAGTCTTCCCCTTTGAATTT-3′
p38	CACCTGTAGGTTCCGGCGCTTACG	5′-AGTCCCGGAGCGATATCAGA-3′	5′-TGGGCATCTATTGCAGAACAAA-3′
XDH1	AAGAATCCGGACGGGAA	5′-AATGAAGCGCCTAAAACCGTATA-3′	5′-TAGGCAGCTGATACCCAATTTTC-3′
XDH2	AAGCAATAATGGCCGCTCGCTCG	5′-GCTTCAGTATTCTTCGCCATCA-3′	5′-CACGGGCACCCCACTGT-3′
Actin	AAGGTTACGCTCTGCCCCACGCC	5′-CTCCCACACCGTACCCATCT-3′	5′-AAGTCGCGACCAGCCAAGT-3′
DJ-1	TTGCTGCCATTTGTGCTGCTCCC	5′-CCACGAGGATAATGGGAAAATC-3′	5′-CCGTGGGCTGCAAACG-3′

Probes and primer sets were custom designed with 5′ labeled 6 FAM™ and 3′ labeled TAMRA.

### Determination of UA Content

Methods for determining UA content were performed using a modification of the method of Tamura et al. [14] The integument (n = 25 each), Malpighian tubules (n = 25 each), testis (wild-type, n = 90; *op*, n = 107) and fat body (n = 25 each) dissected from day 3 fifth instar larvae were pooled to extract UA, and intact hemolymph (n = 12 each) was collected from day 3 fifth instar larvae and stored at -80°C until use. Individual integument (2 mg) was homogenized in 1 ml distilled water (1∶500 volume of water). Pooled tissues were divided into four samples (70 mg each) for fat body, four samples (80 mg each) for Malpighian tubules, and seven samples (70–90 mg each) for testis and samples were homogenized in 0.21 to 0.27 ml distilled water (1∶3 v:v of water). Samples were individually boiled for 10 min and allowed to cool to room temperature. The homogenate was then centrifuged at 10,000×g for 15 min and the collected supernatants were used to measure UA content with a UA Test Kit (Wako, Tokyo, Japan) according to the manufacturer’s instructions.

### Determination of Rotenone LD_50_ in Op Larvae

To determine the LD _50_ of day 3 fifth instar larvae (n = 10 each group) by rotenone (ROT; Sigma) stimulation in wild-type and *op* mutant larvae, we injected 20 µl prepared ROT/g body weight intrahemocoelically to larvae weighing 2.5 to 3.0 g using a disposable syringe (Terumo, Tokyo, Japan) with a 30 G needle. ROT was dissolved in DMSO (prepared immediately before use and stored in the dark) at 0, 1.25, 2.5, 5.0, 10, 20, 40, and 80 µg/g. The number of dead silkworms after 24 h was counted and the mortality rate (%) = (X/Y) × 100 was calculated, where X = dead larvae in the group and Y = total larvae in the group. The mortality rates were analyzed using the JMP 9.0 software (SAS Institute Japan Ltd., Tokyo, Japan) to calculate the LD_50_.

### Two-dimensional (2D) Gel Electrophoresis, and Detection of Oxidized DJ-1

Fat body and testis were dissected from day 3 fifth instar wild-type and *op* larvae for detection of oxidized DJ-1. To prepare total protein extracts for two-dimensional (2D) gel electrophoretic analysis, the tissues were sonicated in rehydration buffer comprising 8 M urea, 2% CHAPS, 0.5% carrier ampholytes at pH 4 to 7, 20 mM dithiothreitol, 0.002% bromophenol blue, and a cocktail of protease inhibitors. Urea-soluble 400 µg proteins were separated by isoelectric focusing (IEF) using the ZOOM IPGRunner system loaded with an immobilized pH 4 to 7 gradient strip (Invitrogen). After the first dimension of IEF, the protein was separated in the second dimension on a 4 to 12% NuPAGE polyacrylamide gel (Invitrogen). For detection of BmDJ-1, the gel was transferred to a polyvinylidene difluoride (PVDF) membrane for immunoblotting using rabbit anti-BmDJ-1 antibody and goat anti-rabbit IgG-conjugated horseradish peroxidase (HRP). The membranes were developed using a chemiluminescent substrate (Pierce, Rockford, IL, USA). Developing time was different between wt and *op* mutant.

### Protein Assay

Protein concentrations were determined using a Bradford assay kit (Pierce, Rockford, IL, USA), according to the manufacturer’s instructions.

### RT-PCR

Total RNA from 500 brains of day 3–5 fifth instar larvae were treated with DNase and processed for cDNA synthesis using primers of 12 to 18 oligo(dT) and SuperScript II reverse transcriptase (Invitrogen, Carlsbad, CA, USA). cDNA was amplified by PCR using Taq DNA polymerase (Qiagen) with the primers for tyrosine hydroxylase (TH; 5′-ACACAACAGTGGTTCAGTCG-3′ and 5′-AGGAGTCTCGCAATGTACAC-3′), actin (5′-TATCGCCGACAGGATGCAGAAGGA-3′ and 5′-TAGAAGCACTTGCGGTGAACGATG-3′) and DJ-1 (5′-CATTTGTGCTGCTTCCATAGCGTT-3′ and 5′-CATTCCCTTTTCGACTTGATCGGC-3′), as described previously [25]. RT-PCR for *B. mori* actin was used as a positive loading control. Amplification was carried out with 28 cycles of denaturation for 40 s at 94°C, annealing for 40 s at 55°C, and extension for 90 s at 72°C. Amplified PCR products were separated by agarose gel electrophoresis, stained with ethidium bromide, and visualized under UV light. The oligonucleotides employed in this study were designed based on the *B. mori* TH nucleotide sequence (Gene ID:100270767), *B.mori* DJ-1 sequence (Gene ID: 100422789)and actin nucleotide sequence (Gene ID: 100145913). Each experiment was performed in triplicate.

### Immunohistochemistry

Serial sections (6-mm thick) were prepared from tissues fixed with 4% paraformaldehyde and embedded in paraffin. After deparaffination, tissue sections were incubated with 3% hydrogen peroxide-methanol for 40 min to block endogenous peroxidase activity. The tissue sections were incubated in a moist chamber at 4°C for 24 hours with anti-tyrosine hydroxylase polyclonal antibody (AB152, Millipore, Billerica, MA, USA) at 1∶1000, followed by incubation with Histofine simple stain MAX-PO (R) for human tissue (Nichirei Biosciences Inc, Tokyo, Japan). The stained slides were developed with ImmPACT^TM^DAB reagent (Vector Laboratories, Inc, Burlingame, CA USA), Mayer’s Hematoxylin solution for nuclear staining (Wako Tokyo, Japan), and examined under the Olympus DP71 universal microscope (Olympus Inc, Tokyo, Japan). Normal rabbit IgG was applied for the negative control.

### Statistics

Mortality was analyzed using probit analysis to calculate LD_50_ levels. Two-tailed Student’s *t*-test was performed in other experiments. All statistical analyses were carried out using the JMP 9.0 software package (SAS Institute Japan Ltd., Tokyo, Japan). For all tests, P<0.05 was considered statistically significant. Data are presented as mean ± SD. Statistical significance was set at *P<0.05, **P<0.01, and ***P<0.001 for comparisons between wild-type and *op* mutants.

## Supporting Information

Figure S1 **Expression profile of each sample of wild-type and** ***op*** **was compared using hierarchical clustering by Ward’s method.** 12,981 probe sets were re-annotated via our annotation pipeline and were used in the analyses.
(TIFF)

Figure S2 ***B. mori*** **TH and DJ-1 mRNA expression in the brain of wild-type and** ***op*** **mutant larvae by qRT-PCR.** Relative mRNA expression in the brain of wild-type and *op* mutants are given as Relative Quantification (RQ) values. RQ represents the relative expression level compared to the reference sample. Error bars represent the relative minimum/maximum expression levels about the mean RQ expression level.
(TIFF)

Table S1 **Gene Set Enrichment Analysis of human homologs in** ***B. mori*** **.** Count is the number of genes associated with the term. The p-values associated for each annotated term inside each cluster have exactly the same meaning/value as the p-values (Fisher Exact/EASE Score) shown in the regular chart report for the same terms [37].
(DOC)

Table S2 **Primers and probes used for quantitative RT-PCR.** Probes and primer sets were custom designed with 5′ labeled 6 FAM™ and 3′ labeled TAMRA.
(DOC)

Table S3 **Complete lists of the re-annotation of Kaiko array probe sets.** Expression values were shown as the log_2_ ratio between wild type and *op* samples (average). Gene descriptions are also shown. The annotated Ensembl transcript ID, the UniProt/SwissProt accession number and gene description for each gene was extracted from Ensembl (release 69) annotations.
(XLS)

Text S1 **Supplementary methods.**
(DOC)

Video S1 **Movie for occasional unique actions such as vibration of** ***B.mori*** **op mutant.**
(MOV)
