# Supplementary figures and images for: Identification of Key Uric Acid Synthesis Pathway in a Unique Mutant Silkworm Bombyx mori Model of Parkinson’s Disease
Source: PLoS One. 2013 Jul 24;8(7):e69130. doi: 10.1371/journal.pone.0069130 (PMC3722175; doi:10.1371/journal.pone.0069130)

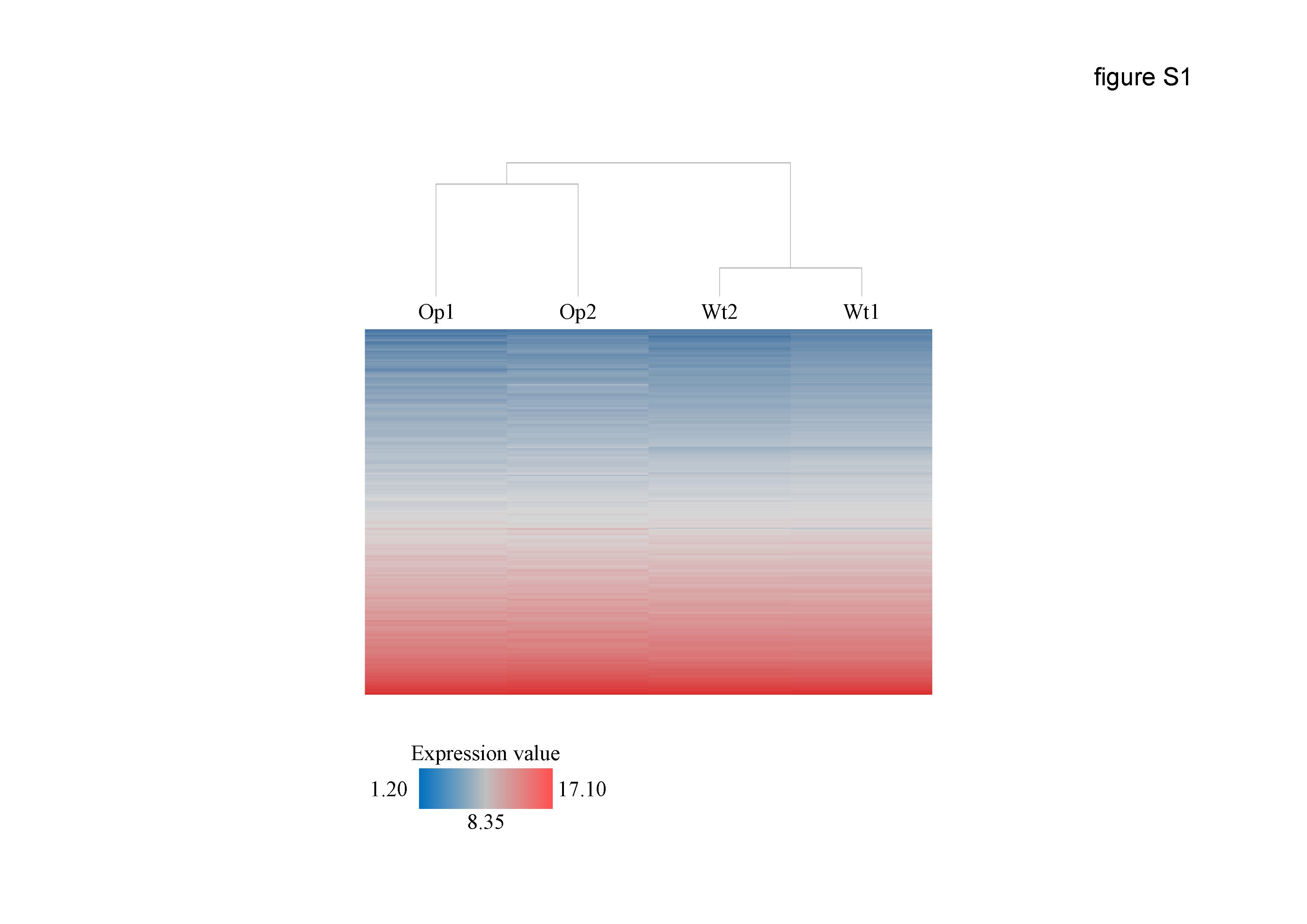

Supplement: Figure S1 — Expression profile of each sample of wild-type and op was compared using hierarchical clustering by Ward’s method. 12,981 probe sets were re-annotated via our annotation pipeline and were used in the analyses. (TIFF) [file pone.0069130.s001.tiff]

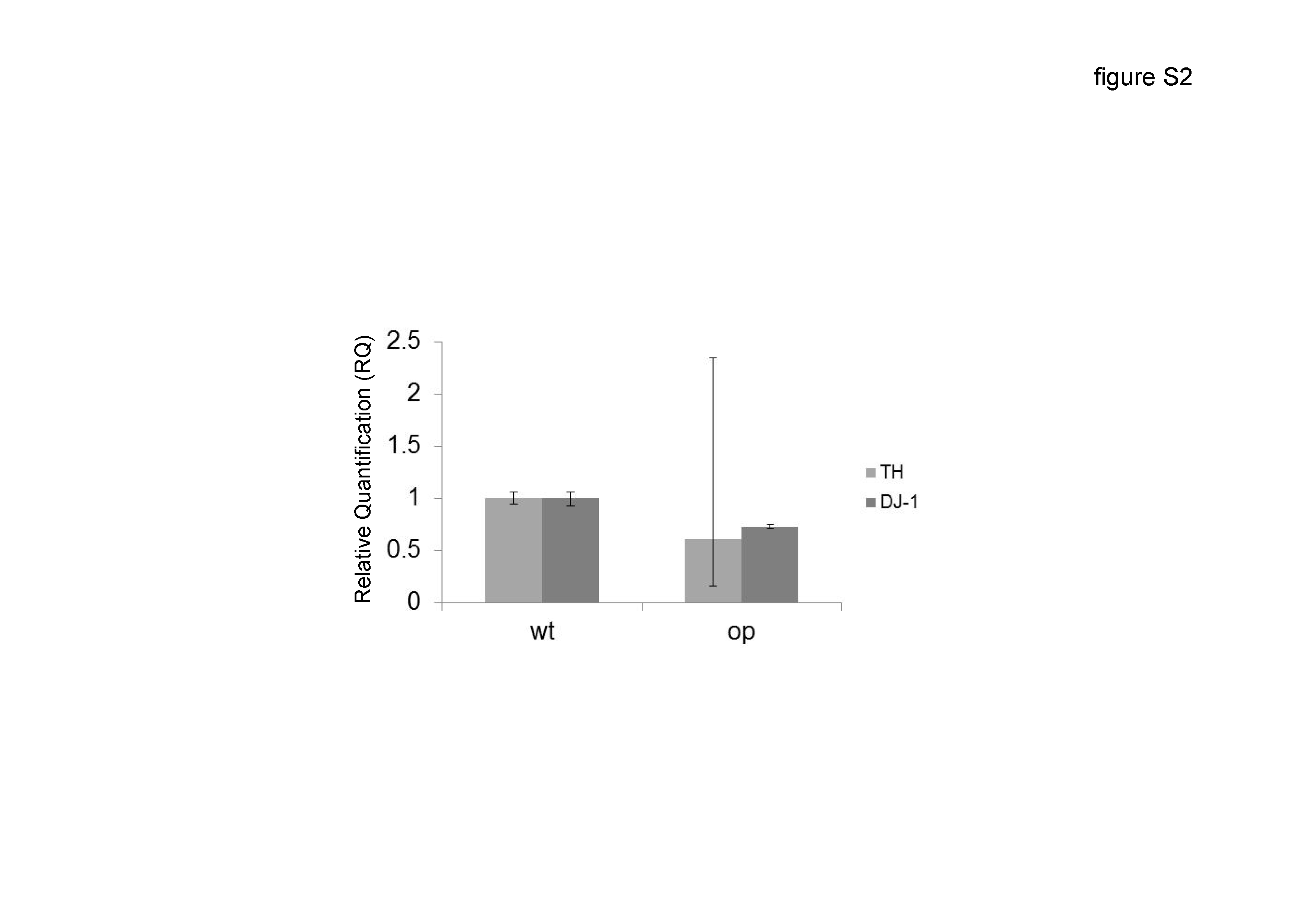

Supplement: Figure S2 — B. mori TH and DJ-1 mRNA expression in the brain of wild-type and op mutant larvae by qRT-PCR. Relative mRNA expression in the brain of wild-type and op mutants are given as Relative Quantification (RQ) values. RQ represents the relative expression level compared to the reference sample. Error bars represent the relative minimum/maximum expression levels about the mean RQ expression level. (TIFF) [file pone.0069130.s002.tiff]
