# Supplementary material for: Identification of Key Uric Acid Synthesis Pathway in a Unique Mutant Silkworm Bombyx mori Model of Parkinson’s Disease
Source: PLoS One. 2013 Jul 24;8(7):e69130. doi: 10.1371/journal.pone.0069130 (PMC3722175; doi:10.1371/journal.pone.0069130)
Supplement: Table S1 — Gene Set Enrichment Analysis of human homologs in B. mori . Count is the number of genes associated with the term. The p-values associated for each annotated term inside each cluster have exactly the same meaning/value as the p-values (Fisher Exact/EASE Score) shown in the regular chart report for the same terms [37]. (DOC) [file pone.0069130.s003.doc]

**Table S1**.

| Pathway | Count | P-Value | KEGG ID |
| --- | --- | --- | --- |
| Spliceosome | 82 | 1.05E-13 | hsa03040 |
| Pyrimidine metabolism | 58 | 2.16E-08 | hsa00240 |
| Purine metabolism | 83 | 3.01E-08 | hsa00230 |
| Aminoacyl-tRNA biosynthesis | 31 | 9.13E-08 | hsa00970 |
| DNA replication | 28 | 1.76E-07 | hsa03030 |
| Ribosome | 49 | 8.06E-06 | hsa03010 |
| Proteasome | 31 | 8.72E-06 | hsa03050 |
| Citrate cycle (TCA cycle) | 22 | 5.51E-05 | hsa00020 |
| Ubiquitin mediated proteolysis | 67 | 6.99E-05 | hsa04120 |
| Glycerolipid metabolism | 28 | 1.19E-04 | hsa00561 |
| Cell cycle | 61 | 1.68E-04 | hsa04110 |
| Huntington's disease | 82 | 2.12E-04 | hsa05016 |
| Drug metabolism | 26 | 4.20E-04 | hsa00983 |
| RNA degradation | 32 | 4.55E-04 | hsa03018 |
| N-Glycan biosynthesis | 27 | 5.92E-04 | hsa00510 |
| Pentose and glucuronate interconversions | 14 | 6.07E-04 | hsa00040 |
