# Supplementary material for: Identification of Key Uric Acid Synthesis Pathway in a Unique Mutant Silkworm Bombyx mori Model of Parkinson’s Disease
Source: PLoS One. 2013 Jul 24;8(7):e69130. doi: 10.1371/journal.pone.0069130 (PMC3722175; doi:10.1371/journal.pone.0069130)
Supplement: Table S2 — Primers and probes used for quantitative RT-PCR. Probes and primer sets were custom designed with 5′ labeled 6 FAM™ and 3′ labeled TAMRA. (DOC) [file pone.0069130.s004.doc]

**Table S2**.

| Gene | Probe | Forward primer | Reverse primer |
| --- | --- | --- | --- |
| Actin | AAGGTTACGCTCTGCCCCACGCC | 5'-CTCCCACACCGTACCCATCT-3' | 5'-AAGTCGCGACCAGCCAAGT-3' |
| DJ-1 | TTGCTGCCATTTGTGCTGCTCCC | 5'-CCACGAGGATAATGGGAAAATC-3' | 5'-CCGTGGGCTGCAAACG-3' |
| TH | TTCGAAGTGCGCTTCAACCCTCACA | 5'-CGCTGGGTGTCAACCATGT -3' | 5'-CAACGGAGTCGAGCACCTCTA -3' |
