# Supplementary material for: Identification of Key Uric Acid Synthesis Pathway in a Unique Mutant Silkworm Bombyx mori Model of Parkinson’s Disease
Source: PLoS One. 2013 Jul 24;8(7):e69130. doi: 10.1371/journal.pone.0069130 (PMC3722175; doi:10.1371/journal.pone.0069130)
Supplement: Text S1 — Supplementary methods. (DOC) [file pone.0069130.s006.doc]

**Supplementary methods**

*Analyzing and visualizing microarray data -* TIBCO Spotfire software (TIBCO, Palo Alto, CA, USA) was used for the identification of differentially expressed probe sets between *op* and wild-type larvae, calculation of correlation coefficients and bi-directional hierarchical clustering by Ward’s method.

*Gene set enrichment analysis for human homologs in B. mori*-BLAST search (program tblastx) was carried out with an E-value threshold of 1e-10, the ‘Consensus gene set by merging all the gene sets using GLEAN’ from http://sgp.dna.affrc.go.jp/pubdata/genomicsequences.html as the query and the whole transcript sequence of human from Ensembl version 68, ftp://ftp.ensembl.org/pub/release-68/fasta/homo_sapiens/cdna/ as the database. By assigning each top hit to a *B. mori* transcript, human homologs were annotated to 8,096 out of a total of 14,623 transcripts.

Utilizing The Database for Annotation, Visualization and Integrated Discovery (DAVID: http://david.abcc.ncifcrf.gov/) v6.7, we performed gene set enrichment analysis (GSEA) to search the functional feature of the transcripts for which homologs could be identified in the human database [37]. GSEA for *B. mori* transcripts was made possible by extracting the human transcript IDs annotated above. Detailed results are shown in Table S1; these indicate that the statistically significant of the metabolic pathway was purine metabolism (p=3.01E-08).

*Quantitative RT-PCR*- To quantify RNA expression levels, total RNA was extracted from pooled brain dissected from day 3-5 fifth instar larvae (n=500 each) using an RNeasy Mini Kit (Qiagen) . Total RNA from 500 brains of day 3-5 fifth instar larvae were treated with DNase and processed for cDNA synthesis using primers of 12 to 18 oligo(dT) and SuperScript II reverse transcriptase (Invitrogen, Carlsbad, CA, USA). RT-PCR was performed in 20 μl reaction volumes using 500 ng of cDNA template and custom-made tyrosine hydroxylase, DJ-1 and actin primers and probes (Table S2) with a TaqMan Gene Expression Master Mix (Applied Biosystems, Foster City, CA, USA) according to the manufacturer’s instructions.

Quantitative RT-PCR (qRT-PCR) was performed on a 7500 Fast Real-Time PCR system (Applied Biosystems) following the Delta-Delta Ct method. Actin was utilized as an endogenous reference against which RNA expression levels were standardized, and all data were calibrated against universal reference data. All assays were performed in triplicate.
